# Dynamics of Nonlinear Systems

**DOI:** 10.1155/2014/246418

**Published:** 2014-12-22

**Authors:** Maoan Han, Zhen Jin, Yonghui Xia, Haomin Zhou

**Affiliations:** ^1^Department of Mathematics, Shanghai Normal University, Shanghai 200234, China; ^2^Department of Mathematics, Shanxi University, Taiyuan, Shanxi 030051, China; ^3^Department of Mathematics, Zhejiang Normal University, Jinhua 321004, China; ^4^School of Mathematics, Georgia Institute of Technology, Atlanta, GA 30332, USA

In the last few decades, theory of dynamical systems has become a rapidly growing area of mathematics and attracted many researchers. This theory is an important and well-established branch of the modern mathematics. The reason lies in the fact that the study of dynamical systems not only has an important theoretical interest, but also is motivated by problems from other disciplines, including physics, chemistry, astronomy, biology, engineering, and many others in natural and social sciences.

With the intention to provide an opportunity for the researchers in the area to publish their most recent findings rapidly, we proposed this special issue. It announced a broad scope on differential, functional, and delayed differential and difference equations, including their qualitative analysis, numerical computations, and applications in science and engineering. The submissions received cover all targeted topics.

There were about 60 manuscripts submitted to this special issue. Each one had been sent to at least two external reviewers for peer review. Based on the reports, we carefully selected 20 original research articles for publication. And they cover a wide range of topics in the general field of nonlinear dynamical systems. Among them, five papers focus on bifurcation analysis on different systems, such as systems with heteroclinic loops, isochronous center, and chaotic solutions; three focus on numerical analysis of symplectic Runge-Kutta method, inexact Newton-Gauss method, and discontinuous mixed covolume methods for ordinary or partial differential equations, respectively; and a few others study different aspects of qualitative behaviors of differential equations, such as limit cycles, asymptotic solutions, and invariant tori. Most of the collected papers study systems of ordinary differential equations; other types are represented too. For example, there are two papers on difference equations, two on singular perturbed differential equations, one on traveling wave problem, and three on delayed differential equations. The published papers cover a wide range of applications as well, including problems from mechanical engineering design, computational fluid dynamics, sliding mode feedback control, thermonuclear reaction, models for infectious diseases, predator-prey system, and financial systems. We hope that readers will find many recent developments reported here interesting.

